# Rural-Urban migration of children and the older population: identifying challenges and adaptive strategies in Iran with a qualitative study

**DOI:** 10.1186/s12877-025-06742-7

**Published:** 2025-11-29

**Authors:** Saideh Garousi, Behshid Garrusi, Faezeh Mohammadi

**Affiliations:** 1 Professor of Sociology, Department of Social Sciences, Faculty of Humanities, Shahid Bahonar University of Kerman, Kerman, Iran; 2 Professor of Psychiatry, Neuroscience Research Center, Institute of Neuropharmacology, Kerman University of Medical Sciences, Kerman, Iran; 3 Researcher, Preventive Medicine and Public Health Research Center, Faculty of Medicine, Iran University of Medical Sciences, Tehran, Iran

**Keywords:** Older adult, Migration, Health, Adaptive strategies, Social support

## Abstract

**Background:**

The phenomenon of population aging and the migration of young people from rural to urban areas, particularly in Iran’s rural regions, has led to a higher growth rate of aging populations. This qualitative study aimed to investigate the effects of children’s migration on the physical, mental, and social health of older adults in Kerman province and to identify their spontaneous adaptive strategies.

**Methodology:**

This study utilized a qualitative approach with thematic analysis as the method. Data were collected through semi-structured interviews with 24 older individuals aged 65 and older who reside in villages in Kerman province. The sampling method was purposive and aimed for maximum diversity, and continued until data saturation was reached. The interviews were conducted over eight months. The validity of the findings was ensured by adhering to the four criteria established by Lincoln and Guba.

**Findings:**

Multifaceted Health Burden, The Collapse of the Multigenerational Support System, The Invisible Emotional Economy, Technology: Temporary Sedative, Cycles of Identity are the primary reconstructions that emerged from the analyses. The findings revealed health challenges in three main areas. Physical Challenges: These included exacerbation of chronic pain, limited mobility, and sleep disorders. Psychological Consequences: Older individuals experienced significant loneliness, anxiety, and reduced self-esteem. Social Support: There was a decline in social support, characterized by the breakdown of multigenerational support systems, gaps in intergenerational caregiving knowledge, and a shift towards one-way communication in relationships.

On the other hand, older people employed various adaptive strategies, such as using technology for virtual communication, redefining social roles, and engaging in local solutions. These included active participation in religious rituals, strengthening local networks, and revitalizing cultural traditions in the absence of their children.

**Discussion and Conclusion:**

The migration of children significantly disrupts traditional support structures for older adults in Iranian families, resulting in a multifaceted health burden for this group. While the adaptive strategies rooted in local culture provide some psychological relief, there is an urgent need to design multilevel support programs. These programs should focus on strengthening local institutions—such as mosques as support centers—developing mobile health services, and integrating national policies with local models. The findings of this study lay the groundwork for policymaking aligned with the World Health Organization’s “Decade of Healthy Aging” goals.

## Background

The aging of the global population presents a significant challenge, with projections indicating that the number of older individuals will double by 2050. In Iran, this trend is progressing rapidly, with the percentage of people aged 60 and over expected to reach 21% by 2050. The growth rate of the older population is notably higher in rural areas (6.82%) compared to urban areas (5.84%). This can be attributed to declining fertility rates, the migration of young people to cities, and the tendency of retirees to return to their villages [1, 2].

Rural-urban migration, driven by economic hardships [3] and climate change [4], has posed challenges to the traditional framework of older adult care. In Iran, 85% of older adults live at home with their families, and out-of-home care is generally not accepted [5].

International studies indicate that children’s migration is linked to contradictory health outcomes. While financial support from migrants can enhance the economic situation of their parents [6], it often fails to offset the increased risk of mental health issues such as depression, anxiety, and loneliness [7–9]. Additionally, it can lead to limited access to healthcare services [10, 11] and a reduced quality of life [12, 13].

Older individuals face numerous challenges when their children are absent. According to the theory of ambiguous loss [14], the physical absence of children, combined with a continued emotional bond, creates a paradox that necessitates special psychological solutions. This challenge is also highlighted in the framework of stress and adaptation theory [15], which suggests that older adults must utilize either emotion-focused or problem-focused coping strategies to manage the stresses of change. From the perspective of Intergenerational Dependency Theory [16], the migration of children can weaken both “functional cohesion” and “affective cohesion” within family networks. In societies where multigenerational support systems are vital for older adult care, such disruptions can have serious consequences. Conversely, the convoy model of support [17] explains how older adults seek alternative resources, such as neighborhood ties, to fill the gaps created by these changes. Additionally, social network theory [18] warns that a reduction in “structural social capital,” resulting from the migration of children, limits access to local support resources and heightens the health vulnerabilities of older persons. These factors indicate the need for a multidimensional planning approach that addresses micro (individual empowerment), meso (strengthening local institutions), and macro (developing national programs) levels.

In Iran’s cultural context, these challenges are intensified by the prevalence of small nuclear families [1], insufficient rural support services [5], and the tension between traditional values and modern life [19]. Despite a wealth of quantitative research in countries such as China [7, 13] and Mexico [11], qualitative studies in the Middle East, particularly in Iran, focusing on migration and left-behind parents are limited [12, 20]. This knowledge gap is especially evident in the area of psychosocial coping mechanisms of older individuals left behind.

To address this gap, the present study adopts an exploratory, open-minded approach (without preconceived assumptions) using qualitative thematic analysis. It pursues two main objectives:

- To explore the consequences of children’s migration on various dimensions of health (physical, psychological, social).
- To discover the methods participants spontaneously employ to face these challenges.

The findings of this study could provide a framework for designing multi-level interventions, emphasizing the World Health Organization’s “Decade of Healthy Aging.”

## Method

### Study Design

The researchers designed this study using a qualitative approach and employed thematic analysis. The study’s objectives included examining the multidimensional challenges and adaptation strategies faced by older adults. Qualitative methods allow researchers to explore complex, multi-layered objectives and to understand cultural and social meanings. The choice of thematic analysis was based on several factors: it is a flexible method that enables an exploration of personal experiences among older people, without the need to adhere to established frameworks. Additionally, its cultural sensitivity allows for the identification of themes unique to the Iranian context [21]. This method can process complexities, especially when faced with contradictory data [22]. Thematic analysis is well-suited to exploratory research. It helps identify new challenges faced by older people in rural areas [23].

### Participants

The study included participants aged 65 or older who lived in villages within Kerman province. The rural population in Kerman is notably high, and the area’s economic and social conditions present several serious challenges. These include a weak rural economy, low income, drought and water supply issues, youth migration, annual household incomes below the national average, and vulnerable infrastructure and services [24]. The selected villages were situated between 45 and 130 km from the provincial center.

For participant selection, the researchers used a purposive sampling method with maximum variation. The first participant in each village was identified with assistance from the rural health center, based on specific entry and exit criteria. Subsequently, each participant was asked to introduce other eligible individuals through the snowball sampling method. The introduction criteria included residence location, age, and varying experiences of child migration. Data saturation was reached after 21 interviews (no new themes emerged), and three additional interviews were conducted for reliability, for a total of 24. In qualitative research, sample size is determined by data saturation rather than statistical representativeness, in line with qualitative methodological standards [23].

Since the study aimed to explore the experiences of older adults rather than to make statistical generalizations, the sampling was designed to achieve maximum diversity. This diversity, including variations in age and experiences related to child migration, helped examine different dimensions of the phenomenon.

The interviews involved 24 participants: 18 women and six men. The imbalance in the gender ratio arises from limited access to older rural men, who are often engaged in agricultural or livestock work and have less time available for interviews. Furthermore, men may be less willing to disclose feelings such as loneliness or depression, which are crucial for qualitative interviews. In Iran, rural men have a lower life expectancy than women [1], so the number of male participants is lower. Even with snowball sampling, attracting more men proved challenging. Nevertheless, the experiences and quotes of male participants were included in the findings.

The inclusion criteria for participants included: being 65 years of age or older (verified with the village health center liaison. This age was by the standards of aging studies in Iran [2]. Having permanent residence in the village for the past year. Participants were also selected based on their living situation—alone or with a spouse—to minimize the impact of support from other family members on their daily lives. Functional independence was another important criterion; each participant’s ability to dress, bathe, feed, transfer, toilet, and control urination/defecation was assessed by the health center liaison. Only those who demonstrated functional independence were included in the interview phase to exclude caregiver-dependent older adults who might have different experiences [25].

Three exclusion criteria were established: participants were excluded if they had any communication barriers that interfered with verbal responses (e.g., dementia, aphasia, hearing loss, or inability to speak); if they had not utilized at least one support service from a local NGO, welfare organization, or relief committee since their child’s migration (to ensure that no alternative support networks influenced their experiences); or if their last surviving child had migrated less than 12 months prior to the study (to eliminate the short-term effects of migration shock [26].

### Data collection

The interviews were designed in two phases. The preliminary phase involved three unstructured exploratory interviews with open-ended questions. The central questions during this phase were: “How would you describe the changes in your daily life after your children migrated?“, “What new challenges have you faced after living alone?“, and “What methods have you used to cope with the migration of your children?” The results from this phase informed the design of the semi-structured interviews in the second phase. To prepare the interview questions, we followed three stages:

1. A systematic review of the literature regarding older individuals whose children have migrated.
2. Establishing a focus group that included a social psychiatrist, a sociologist, and a village doctor.
3. Conducting pilot interviews as a practical test.

We carried out three pilot interviews with older individuals from rural backgrounds (outside the main sample) to assess the comprehensibility and appropriateness of the questions. This assessment involved agreement among the coders and group discussion. A sample of the research questions is presented in Table No. 1.

**Table 1:** Sample interview questions

scope	Main questions	Follow-up questions
Health self-assessment	Did your physical health change after your child immigrated? Please explain more. Did your mental health change after your child immigrated? Please explain more.	Has your sleep changed at night? If so, what do you do to improve it?
Key challenges	Have you had any problems in your life since the kids left?	“Can you tell me about some times when you needed help from your child but didn’t?”
Adaptation methods	“Can you tell me what you did to cope with loneliness/problems?”	““Have you tried to increase your contact with your children?”
Consequence	What impact do you think the children’s migration has had on your life?	““Do you feel like the relationship has become closer or more distant? Do you feel like they still love you as much as before?”

In the second phase, the interviews lasted between 45 and 90 min, with an average of 67.5 min. All interviews were conducted by trained researchers, specifically by a team of three interviewers (two women and one man) in locations suggested by the participants, such as their homes or rural health posts. The interview timings were pre-approved by the village health center contact, and the interviews were scheduled during quiet periods to ensure participant privacy and good audio quality. The research team conducted all interviews over 8 months.

Audio recordings were made with written consent obtained at the beginning of each interview. Coding was performed concurrently with data collection, which significantly aided in the purposive selection of subsequent participants based on emerging themes. Theoretical sampling, as recommended by Charmaz [27], was also employed.

To ensure participant privacy, we obtained written consent before the interview for audio recording and the publication of anonymized data. All identifying information (such as names, addresses, and contact details) was replaced with codes (e.g., P1, P2). The audio and text files of the interviews were stored securely, accessible only to members of the research team. After the research concluded, all audio files were destroyed. During the interviews, only the interviewer and the participant were present; for interviews with male participants, only the male interviewer conducted the session in the presence of a team member.

### Steps for Conducting Interviews

1. Identifying Eligible Participants: Participants were contacted through the village health center to provide a general introduction to the study and schedule an interview time and location. They were informed that the interview would likely last more than an hour.

4. Introduction: The interviewer began with an introduction: “Hello, I am [name] from Kerman University of Medical Sciences. Our research focuses on the experiences of older adults after their children migrate.”
5. Objectives: A clear statement of the research objectives followed: “This study aims to understand your challenges and solutions so that policymakers can provide better services.”
6. Ethical Principles: The ethical principles of the study were outlined, emphasizing the voluntary nature of participation, the right to withdraw at any stage, confidentiality of information, and access to the final results. Participants signed an informed consent form granting permission for audio recording and publication of anonymous quotes.
7. Conducting the Interview: According to the interview protocol, the researcher began with non-sensitive questions, such as inquiries about daily activities. Standard probes such as “Can you explain more?” and “Can you provide an example?” were used. Interviewers were required to note non-verbal behaviors, including laughing, crying, or frowning.
8. Managing Interaction: Interviewers adapted their language to match the participant’s literacy and understanding levels. Short breaks were incorporated as needed, and active listening was emphasized, with interviewers confirming understanding by stating, “If I understood correctly, do you think…?”
9. Quality Control: To maintain the quality of the interviews, interviewers avoided leading questions and were trained to halt the interview if the participant displayed signs of emotional distress. At the end of each interview, the interviewer summarized the responses to confirm accuracy with the participant. Finally, the interviewer asked, “What is the most important thing you wanted to say that you did not mention?” Participants were thanked for their time, and the researcher’s contact information was provided to all.

The consistent implementation of this protocol helped stabilize the findings. The project supervisor and the ethics committee regularly reviewed interviews to enhance the validity and ethical monitoring of the research.

Increase the validity and ethical monitoring of the research.

### Validation of Findings

We used four strategies based on the Lincoln & Guba [28] framework. 1. Triangulation: Independent coding was performed by two experts (first person: sociologist/second person: psychiatrist). Then, the codes were compared in several stages. Group discussion was used to minimize differences. This process continued until an acceptable agreement coefficient was reached.

10. Participant review: Modification of themes based on participant feedback. Providing a summary of the findings to 7 randomly selected participants and collecting their feedback led to modifications to some themes. At this stage, the participant was explicitly asked whether the interpretations were consistent with their experience.
11. External audit: Confirmation of the process’s compliance with quality standards by an independent observer. One of the professors from Shiraz University, an expert in this field, reviewed the entire work and coding process in accordance with qualitative research standards.
12. Rich description: Providing contextual details and direct quotes. Direct quotes were used in the final research report, with participant codes (not actual identities) mentioned. A detailed description of the village context was provided to outline the research context. Moreover, any inconsistencies observed were noted in the final report. This approach ensures (eliminating interpretive bias) and a rich description for judging applicability to other contexts.

### Ethical considerations

This study followed research ethics. It got approval from the Kerman Medical University Ethics Committee (IR.KMU.REC. 1400/711). Before the interviews began, the researchers obtained written informed consent from all participants (for participation and publication details). The researchers provided participants with a detailed explanation of the research objectives, the participation process, and the interview length. They were also informed about their right to withdraw without facing negative consequences. We promised them at the end of our talk that their information would stay private. To keep privacy, we removed all identifying information. They got clear answers to their research questions. Also, they accessed the final results. No one applied financial or psychological pressure to encourage participation. The researchers held the interviews in a calm, stress-free setting. They protected participants’ privacy when using public places such as health centers.

Clinical trial number: not applicable.

## Results

### Interview Participant Characteristics

The age distribution of the participants was as follows: 65–70 years (*n* = 6), 71–80 years (*n* = 15), and over 80 years (*n* = 3). In terms of education level, the distribution was: illiterate (17 individuals), primary education (4 individuals), and diploma holders (3 individuals). Some participants had mixed sources of income, including agriculture, pensions, and weaving. Notably, 79% of the participants were economically inactive. 92% lived in homes they owned or their children owned, while 8% were tenants. It is important to note that women had a higher average age, and 5 participants were widows. On average, participants reported having four children and described their incomes as low to moderate.

### Analysis of What Has Been Discussed

Data analysis was carried out using qualitative thematic analysis. In the first phase, open coding was employed to scrutinize each part of the interview, identifying and labeling significant concepts, yielding 100 initial codes.

After reviewing these codes, similar items were grouped, yielding subthemes. Subsequently, these subthemes were merged based on their similarities, resulting in five main research themes. Multifaceted Health Burden, The Collapse of the Multi-Generational Support System, The Invisible Emotional Economy, Technology: Temporary Sedative, Cycles of Identity are the primary reconstructions that emerged from the analyses. Each is explained below, along with its sub-themes. This reflects the core challenges and adaptive strategies identified among older rural Iranians affected by children’s migration. For detailed examples or key codes under each sub-theme, refer to Table 2 in the manuscript.

**Table 2:** Thematic analysis of interviews

Main themes	Sub-themes	key codes	Frequency
1. Multifaceted health burden	1.1Physical Problems	• Exacerbation of chronic pain	21 people (88%)
		• Decreased physical activity	18 people (75%)
		• Sleep disturbance	16 people (67%)
	1.2Psychological Challenges	• Feeling lonely	23 people (96%)
		•Recurrent negative thoughts	20 people (83%)
		•Low self-esteem	17 people (71%)
	1.3 Lack of Care	• Delay in receiving treatment	19 people (79%)
		• Self-medication•	14 people (58%)
		• Malnutrition	12 people (50%)
2. The collapse of the multi-generational support system	2.1 The Chain of Care Break	• Visiting a doctor, unaccompanied	18 people (75%)
		• Discontinuation of medication monitoring	5 people (63%)
		• Neglect of medical follow-up	10 people (42%)
	2.2 The Inter-generational Care Knowledge Break	• Unused indigenous medical knowledge	15 people (63%)
		• Changing cultural values ​​in care	13 people (54%)
3. The invisible emotional economy	3.1 Unproductive Investment	• Unrequited love	22 people (92%)
		• One-sided support	17 people (71%)
		• Memories without heirs	14 people (58%)
	3.2 Inflation of Unrequited Emotions	.Unanswered messages	16 people (67%)
		• Long waits	11 people (46%)
		• Unrequited motivational efforts	11 people (46%)
4. Technology: Temporary sedative	4.1 Inadequate Communication	• Short calls	16 people (67%)
		• Difficulties expressing feelings	12 people (50%)
		• Time limits on contact	10 people (42%)
	4.2 Luggage Care	• Temporary houseguests	14 people (58%)
		• Intensive care	13 people (54%)
		• Emotional burden of visits	11 people (46%)
5. Cycles of identity reconstruction	5.1 Reflexing Roles	• Supportive parent	14 people (58%)
		• Grandparent	12 people (50%)
		• Playing the patient role	9 people (38%)
	5.2 Rethinking	• Redefining value	12 people (50%)
		• Worship	15 people (63%)
		• Adjusting expectations	13 people (54%)
	5.3 Native Solutions	• More contact with villagers	13 people (54%)
		• Redefining traditions in the absence of children	8 people (33%)

Due to limitations, only key codes are outlined in this manuscript. Table 2 presents examples of initial codes, subthemes, and main themes.

## Multifaceted health burden

### Physical problems

Most participants reported a variety of physical problems categorized into three areas: exacerbation of chronic pain, decreased physical activity, and sleep disorders. These challenges were so prevalent that 88% of participants reported an increase in chronic pain, while 67% noted disturbances in sleep (Table 2). These issues severely impaired their quality of life. For instance, Participant 3 shared, “When I wake up in the morning, I feel like I’ve been up all night. My body aches, and it takes me about an hour to get out of bed and prepare breakfast. I’ve become very slow. Before, when my daughter lived with me, I didn’t understand these problems at all.”

### Psychological challenges

Feelings of loneliness, frequent negative thoughts, and low self-esteem were common themes in the interviews. Many older participants expressed that the absence of their children engendered a profound sense of homesickness. They reported concerns about anxiety and depression, attributing their mental health issues largely to loneliness. Loneliness was identified as the primary psychological challenge faced by 96% of older adults, while 83% reported experiencing frequent negative thoughts(Table 2). Participant 20 remarked, “It’s been almost 15 years since my children left home one by one. None of them lives in the village. Only my wife and I are at home. The children come to the village a few times a year. I can tell you with certainty that almost all parents and acquaintances are in the same situation. They are alone.”

### Lack of care

Participants emphasized the need for physical care, stating that due to age, age-related diseases, and physical limitations (e.g., walking, vision, and hearing), they required assistance from their children. In their absence, many faced significant challenges. For instance, Participant 12 mentioned, “I do not have good vision. I cannot walk properly. I wanted to go to the yard; I fell and broke an arm and an ankle. There was no doctor, my wife took care of me until the morning, a neighbor came and took me to the clinic, and in the afternoon, my son came and took me to the city, where I received treatment.”

Due to a lack of companionship or support, many older adults resorted to unprofessional treatments or even experienced malnutrition. Common examples of self-medication included using previous prescriptions, sharing medications with others, or relying on herbal remedies. Participant 6 stated, “I couldn’t sleep at night. My sister gave me her pills. I was fine for two nights. Then I almost died. The doctor said it was a drug interaction.” Malnutrition at 50% and self-medication at 58% illustrate a failure in care (Table 2).

## Collapse of the multigenerational support system

### Gap in the chain of care

In Iran, multigenerational families and close intergenerational relationships have traditionally played key roles in the care of older people [29]. However, the migration of children has weakened or completely disrupted this chain of care. Many participants noted that they often carried out daily tasks and medical visits without the support of their children, resulting in serious ongoing care issues. This gap in caregiving not only affects the physical health of older adults but also leads to feelings of helplessness. Participant 19 shared, “I lost my medications. When I go to the doctor, I forget what to tell him. If my son were with me, I wouldn’t feel so embarrassed.” This gap in care was evident in several ways, such as only seeing a doctor (75%) and failing to monitor medication use (63%) (Table 2).

### Disruption of intergenerational caregiving knowledge

Another significant aspect of the traditional Iranian family is the intergenerational transmission of care, indigenous knowledge, and experiences, which children’s migration has disrupted. This knowledge encompasses traditional caregiving methods, nutritional practices, and the use of indigenous plants, typically passed down through generations. Participant 13 recounted, “When I was a child, my grandfather and maternal aunt, who were older, lived with us. I learned from my mother and other women in the family how to care for older people, change their clothes, and feed them. But now, my children don’t know what to do with me.” 63% of participants noted the underutilization of indigenous medical knowledge, while 54% highlighted shifting cultural values in care (Table 2).

## Invisible emotional economy

### Unproductive investment

In this subtheme, many older adults feel that the love, time, and care they invested in their children throughout their lives have been wasted after their children emigrated. This experience contradicts traditional Iranian family values, which emphasize the expectation of emotional return and support from the younger generation [31]. P 7: “I spent my whole life on my children, but now they only come to visit me on Nowruz.” The sense that their love and support felt one-sided was reported very frequently (92%) among older participants in the study (Table 2).

### Inflation of unproductive emotions

Numerous older adults report heightened emotional expectations paired with a decrease in reciprocal affection. They frequently find themselves waiting for their children’s calls, messages, or attention, but these communications have become less frequent and more superficial due to work or cultural obligations. Feelings of rejection and damaged self-esteem often result from this disconnect. P9: “I am waiting for a call or message, but most of the time there is no news.” Waiting for a call or message without a response (67%) and unreciprocated motivational efforts (46%) were examples of this emotional inflation (Table 2).

In Iran, deep emotional ties between generations are integral to the family’s identity and social structure. The migration of children has not only weakened the support system but also disrupted the acculturation process within families. These findings differ from societies where family relationships are primarily based on welfare functions [32], rather than emotional bonds.

## Technology: temporary sedative

### Insufficient communication

In this subtheme, many seniors express that their telephone or virtual communication with children is often limited and inadequate. They indicate that this brief communication may alleviate feelings of loneliness but cannot replace deep and meaningful relationships. For 67% of older people, communication was limited to short calls, and for 50% of them, expressing true feelings was difficult(Table 2). P11: “I call my son on my mobile phone. He talks very quickly so that he can return to work sooner. If I call him in the morning, he is in the office and cannot easily answer me. If I call at night, he is busy with his children. It’s nice to feel somewhat connected, but I can’t easily share everything with him” [33].

### Luggage care

This subtheme refers to the short-term and temporary in-person visits children make. Although seniors appreciate these visits for the immediate support they provide, this support is not permanent or sustainable. Visits often come with pressures and discomforts for older adults, such as the journey to the city and unfamiliarity with a new environment. P15: “Sometimes I go to my daughter’s house in the city. It’s nice to see them and be close, but I don’t feel comfortable in a strange city or my daughter’s house. I wish I could return home sooner.” P23: “I still hold mourning gatherings during Muharram, just like before. I cook traditional food, and all my children come to my house with their families. It’s wonderful when we’re all together. I’m happy. But they will return soon. They have to go to school, university, and the office.” For 67% of older adults, communication was limited to short calls, and 50% found it difficult to express their true feelings (Table 2).

These short and temporary connections present a new challenge for older adults in Iran, where family is the primary support network [34].

## Cycles of identity reconstruction

### Reflective roles

After their children migrated, older people faced a reassessment and redefinition of their roles within the family and community. Some accepted the responsibility of caring for grandchildren temporarily, viewing this role as an opportunity to remain actively involved and to maintain their status as parents or grandparents. This redefinition of roles was seen in forms such as “supportive parent” (58%) and even “adopting the patient role” (38%) to receive attention and protection (Table 2). P22: “In the summers, one of my grandchildren, who is 5 years old, comes to stay with me. I feel like a mother to her.” Others, drawing from their life experiences, have become references for family dispute resolution or counseling their children. P6: “After all, we are older and have experience. We need to help the children. Whenever my son and daughter-in-law fight, I try to mediate. I talked to them so much that, after their divorce, they reconciled and remarried. I even asked my daughter-in-law to come back.”

Some older individuals acknowledge that they sometimes play the role of a sick person, either consciously or unconsciously, to encourage visits from their children or relatives. P12: “Sometimes when I feel sick, I’m happy because it means one of the children comes to visit me.”

This variety of role-playing demonstrates the adaptation of Iranian older people to changes in the family. Redefining roles after children’s migration is not only a sign of older people’s adaptation and psychological strength, but also an active effort to sustain their identity, status, and value within the socio-familial context. Recent studies highlight that maintaining an active role in family and society is crucial for mental health and a sense of purpose during old age, even as family structures rapidly evolve [35].

### Rethinking

This subtheme concerns the process by which older adults revise their expectations for their children and their own lives. They have adjusted their expectations regarding their children and their own lives. They have adapted their outlook by believing that circumstances will change and by accepting the limitations that come with old age. Rather than concentrating on their separation from their children, many have turned towards strengthening their spiritual connections through prayer and worship, working to better tolerate their situations through adaptation.

One participant stated, “I told myself that instead of feeling sad, I should read the Quran every day and send its reward to the deceased members of my family.” Redefining the value of life (50%), adjusting expectations (54%), and turning to worship (63%) were key strategies for older adults in this rethinking.

### Indigenous solutions

Older individuals have actively embraced indigenous and local solutions to combat loneliness and the lack of support. These strategies include engaging in local conversations and redefining family and community traditions in the absence of their children. Such solutions help maintain social ties. For example, one participant shared, “I socialize with other people of my age. We sit together, drink tea, and reminisce. I used to be a teacher. Now people are encouraging me to become a member of the village council, and I don’t think that’s a bad idea. I can manage this role.”

Redefining traditions in the absence of children is one of the indigenous strategies implemented by older people to maintain social and cultural connections, particularly amid children’s migration. In this process, older persons strive to uphold cultural continuity by adapting the ways in which family and local rituals and ceremonies are performed. Some older adults believe that customs, traditions, and rituals should be preserved even after their children have emigrated, even though contemporary practice may differ from what it was before migration. The primary objective is to maintain their cultural and social identities.

This ongoing process bolsters the sense of belonging among older people and helps reduce feelings of loneliness and isolation. For instance, one participant mentioned, “I used to go to the mosque for congregational prayers. Now that I am alone and have difficulty walking, and with the children not around, a neighbor comes and takes me to the mosque on his motorcycle. I pray, talk to others, and meet people.” Or P17: “I used to cook food for the Arbaeen and distribute it to the whole village. People would even come from surrounding villages to help prepare the food. Now that I have no one to help me, I only cook a small amount —just enough for our alley. It’s for Imam Hussein; I cannot let this ceremony go unobserved.”

Family customs and traditions place significant emphasis on emotional connections and identity bonds, and redefining these traditions is a crucial strategy for preserving these bonds in the face of social change [36]. The identity of older adults is constantly redefined in interaction with social and family roles [37].

## Discussion

Migration of children to cities, as a critical stressor, has put serious pressure on the physical, mental, and social health of older people in rural Iran. In this study, the physiological burden experienced by older adults, including worsened chronic pain, mobility limitations, and sleep disorders, alongside notable psychological stress arising from emotional loneliness and decreased self-esteem. A distinctive aspect of this health burden in Iran is the ongoing environmental crises of drought and water scarcity, which have severely disrupted agricultural livelihoods and disproportionately impacted older persons. In this context, loneliness does not merely stem from the absence of companionship; rather, it becomes more pronounced during livelihood crises and periods of environmental instability [19, 38, 39]. Given the drought crisis affecting the Middle East, particularly in some neighboring countries, it is anticipated that rural older populations in parts of the region will encounter similar challenges.

According to stress and coping theory [15], an individual’s perception of stressful events and their ability to cope determine the intensity of psychological stress. Coping strategies can be categorized as problem-oriented (focused on changing the situation) or emotion-oriented (focused on managing emotions). The research findings clearly illustrate the connection of this theory to the experiences of older people. Some older adults have attempted to improve their circumstances by increasing social interactions with peers in the village, participating more actively in community events, and seeking support from local institutions such as mosques. Others have managed their negative emotions by accepting their new circumstances, emphasizing spirituality (through prayer and recitation of the Quran), or redefining their relationships with family and life (emotion-focused coping). It is common for older adults to employ both coping strategies simultaneously, such as socializing more with neighbors while also finding solace in prayer.

Bowen’s family systems theory [40], the family as a dynamic system maintains intergenerational interactions to establish balance. However, the migration of children disrupts this balance, removing older people from multigenerational support networks. This structural break makes individuals’ psychological and physical reactions more pronounced. The reduction of emotional and social support evolves beyond mere loneliness into a significant lack of local and multigenerational support networks, leading to increased vulnerability among older adults [41, 42]. In response, older adults use individual strategies, such as redefining their identity roles, actively caring for grandchildren, and participating in religious rituals to enhance psychological resilience and adaptation [43, 44].

The findings concerning the health burdens experienced by older adults are consistent with studies conducted in countries such as China and Mexico [7, 11, 13]. However, an in-depth examination of the Middle Eastern context uncovers critical differences influenced by socio-economic and cultural structures.In comparison with neighboring countries, it is evident that the lack of complementary support networks and the severity of environmental and economic crises in Iran have significantly deteriorated the psychophysical health of older people [45, 46]. In countries like Lebanon, for instance, extended family networks and robust tribal or community ties often serve as a flexible safety net for older adults [47, 48]. In contrast, Iran has undergone a more rapid and pronounced shift toward nuclear family structures, driven by significant economic pressures, escalating environmental crises, including prolonged droughts [24, 39], and high rates of rural-to-urban migration [49].

Despite the economic difficulties faced in Lebanon, the multigenerational family remains both a cultural ideal and a practical reality, thereby offering protection for older adults against isolation [47]. Conversely, in the rural Iranian context of this study, the migration of children frequently culminates in the disintegration of the primary support system for older adults. Furthermore, unlike some neighboring nations, Iran’s formal support systems and rural healthcare infrastructure are less developed, resulting in a substantial gap in family support [5, 37].

Extensive research indicates that, despite rapid globalization, the extended family continues to play a crucial role in the Middle East, providing strong support networks for its members [50, 51]. Even migrants from other Muslim communities tend to rely on their extended families for the care of older adult relatives after relocating [52]. The interplay of diminishing traditional structures and inadequate institutional support creates a unique vulnerability for older adults in rural Iran, rendering their circumstances particularly acute. Although child migration is a widespread phenomenon throughout the region, implications are more significant in Iran due to the specific interactions among accelerated nuclear family formation, environmental pressures, and insufficient public support frameworks.

The present study indicates that when faced with unequal emotional investment, rural older individuals employ emotion-focused coping strategies, such as prayer, participation in religious rituals, and role redefinition, to manage psychological tensions. These strategies are part of an active process aimed at coping with feelings of hopelessness and a lack of mutual support. Additionally, older individuals may attempt to attract attention and support by adopting the role of the “patient.” This can be understood through Parsons’s patient role theory [53] and Goldner’s [54] norm of reciprocity. Parsons believes that when a person plays the role of “sick”, they are temporarily exempted from some social responsibilities and roles and seek care and support from others. In this study, rural older people who feel asymmetric support and loneliness sometimes try to attract the attention and support of their children or those around them by playing the role of “sick actor”. They may seek to rebuild their support network and reduce their psychological stress by showing weakness or need.

The theory of reciprocity emphasizes that social relationships are formed based on mutual exchange and balance of interests and behaviors. When this balance is disrupted—for example, by one-sided emotional investment—an individual may feel worthless and stressed. In this study, older adults experience a decline in mutual support from their children, which reinforces feelings of loneliness and emotional exhaustion. Therefore, they attempt to restore the support balance through emotion-oriented coping strategies, such as prayer and participation in religious ceremonies, alongside role redefinition (assuming the sick role).

While new communication technologies, such as telephone calls and messaging apps, can provide temporary relief, they cannot replace face-to-face interactions and deep emotional support due to older adults’ limited digital literacy, infrastructure limitations, and poor communication quality. This phenomenon has been analyzed as “technological loneliness [55], which describes a superficial sense of connection coupled with profound psychological loneliness. Digital inequality [56] further exacerbates the situation, resulting in increased social isolation among older adults. In rural Iran, digital inequality manifests as a lack of access, skills, and opportunities to utilize digital technologies, limiting their ability to benefit from communication tools and electronic services. It is important to recognize that several factors hinder effective communication in rural areas. These include inadequate internet infrastructure, limited digital literacy with complex applications, and access restrictions, such as internet filtering. One participant (P18) highlighted these challenges: “The internet in the village is always disconnecting and reconnecting. I have tried video chatting with my grandson several times, but it never connects. I do not understand these applications at all.” This digital inequality [56] worsens the situation, leading to increased social isolation among older adults.

According to the communication richness theory [57], technological interactions often fail to convey complex emotions due to diminished message quality and richness. Studies conducted in Morocco [58], Jordan [59], and other Arab countries [60] have shown that digital literacy training and local content production can reduce these gaps; however, this area has received less attention in Iran.

One prominent theme identified in the data is the emergence of one-sided family relationships following children’s migration. Older people reported that, despite their significant efforts to provide material and emotional support to their families and to care for their assets and grandchildren, they have experienced a considerable decrease in the mutual support they receive. This situation arises from geographical distance, changing roles, and reduced regular contact. As a result, these one-sided relationships exacerbate feelings of loneliness, worthlessness, and emotional exhaustion, making older people more vulnerable to physical and psychological challenges.

In the context of identity redefinition and reconstruction, older adults create new social and psychological roles—such as active parent, grandchild supporter, social participant, or patient—to maintain their sense of meaning and psychological coherence. This can be framed within the theories of symbolic interaction and psychological capital [61]. According to symbolic interaction theory, an individual’s identity is formed through social interactions and continually redefined and reconstructed as individuals interpret and make sense of their social situations and various roles. In this study, older persons seek to maintain a sense of meaning and psychological consistency by adopting new social roles, thereby redefining their position in society and adapting to life’s new realities.

Psychological capital theory emphasizes the positive psychological resources that individuals possess to cope with stress, including hope, optimism, resilience, and a sense of personal adequacy. Findings indicate that older people enhance their psychological capital by engaging in religious and social activities, such as participating in Muharram and Arbaeen, celebrating Nowruz, and attending mosques. These activities serve as spiritual and social resources that bolster their hope and resilience.

Participants frequently acknowledged the role of Muharram mourning ceremonies (including chest-beating, visiting Imamzadeh, baking offerings, and reciting prayers and religious poetry) in fostering social support, strengthening collective identity, and enhancing a sense of belonging. This ceremony has been observed in urban and rural areas of Iran for 1,400 years, and the Arbaeen ceremony fits into this pattern as well. Moreover, the Nowruz celebration, marking the beginning of the Iranian New Year, further cultivates a sense of belonging, social solidarity, and happiness through social visits and relationship building. Additionally, mosques serve as crucial gathering places for older adults in Iranian villages, providing further community support and attempting to cope and adapt through confrontational strategies and by redefining their roles. However, the lack of institutional support and the breakdown of the multigenerational system add further pressure, highlighting the cultural and environmental differences between Iran and other countries.

Despite the imbalance in gender sampling our thematic analysis found no significant differences in challenges, such as loneliness and lack of access to care, or coping strategies, like redefining roles and using technology, between male and female participants. This suggests that the experience of losing a multigenerational support system transcends gender. Despite the unbalanced sample, the themes identified likely reflect the everyday experiences of older adults in rural areas.However, given the limited number of men, the results should be interpreted with caution.

### Research Limitations

While this study analyzes the consequences of children’s migration on rural older adults, it has several important limitations that should be acknowledged when interpreting the results and generalizing the findings to a broader population:

13. Small Sample Size and Gender Imbalance: The research sample included 24 participants, of whom 18 were women and 6 were men. Although sampling continued until theoretical saturation was achieved, the relatively small number of men may not adequately represent the experiences and attitudes of male older adults. Although the initial analysis did not reveal a significant difference between the two groups, the small sample size of men may have hindered the ability to identify potential gender differences. Therefore, it is recommended that future studies revisit these findings using a gender-balanced sample.
14. Geographic Limitations: The study was conducted solely in Kerman province, which has specific environmental conditions, including widespread drought and high migration rates. These regional characteristics may have influenced the findings, making them less applicable to other rural areas in Iran or across the Middle East. Social, economic, and environmental differences in other regions could yield different consequences for older people, which were not explored in this study.

### Research suggestions

The findings of this study emphasize the need for multi-level, eco-friendly support programs to alleviate the health burden on the neglected older population. We propose the following actionable recommendations:

15. Mobile health units: Establish mobile health teams of doctors, nurses, and social workers to provide home visits, vaccinations, and medication distribution in remote villages.
16. Digital literacy training: Create workshops at local venues like mosques and libraries to teach older people how to use messaging apps, video calls, and search for health information online.
17. Mosques as support centers: Transform mosques into centers providing social and health services for older persons, including exercise classes, support groups, and psychosocial counseling.
18. Telehealth Integration: Develop a low-cost telehealth system allowing rural older adults to consult with healthcare providers via phone, supported by local health workers.

Successful implementation requires national commitment, collaboration among various ministries, and active involvement from the community and older people. Investing in these programs will improve seniors’ quality of life and reduce long-term medical costs.

### Future Research Directions

Future studies should address the identified gaps by focusing on several key areas:

19. Conducting longitudinal studies to examine the medium- and long-term effects of child migration on the health of rural older individuals.
20. Analyzing the role of digital literacy training and communication technologies in reducing technological loneliness and strengthening emotional connections among older people.
21. Conducting local qualitative research that emphasizes cultural coping strategies, religious participation, and the redefinition of older adults’ identities.
22. Evaluating the effectiveness of support interventions and developing innovative programs to enhance the quality of care, along with regional and international comparative studies to explore differences and similarities in challenges and solutions, particularly concerning environmental and economic factors.
23. Future research should focus on creating and assessing a Local Action Plan that directly incorporates and applies the findings of this study regarding older adults’ self-adaptation strategies, such as the roles of mosques, local networks, and the redefinition of rituals. This plan should align with Iran’s National Action Plan for the World Health Organization’s “Decade of Healthy Aging.” Collaborating with key institutions, such as the Ministry of Health and the Welfare Organization, could facilitate the design of intersectoral interventions that leverage existing socio-cultural resources and address the specific needs of older adults in rural areas affected by migration.

## Conclusion

Children’s migration presents a multifaceted challenge for rural older adults in Iran, encompassing physical and psychological burdens, the collapse of multigenerational support systems, and limited access to technology. This situation results in loneliness, reduced access to healthcare, and a decline in quality of life. The significant role of social, cultural, and institutional supports—such as religious ceremonies, mosques, and family structures—is evident in their role in mitigating these issues. Older adults draw on their psychological resilience through indigenous strategies, such as participating in rituals and fulfilling spiritual roles. Therefore, developing local support systems and promoting digital literacy should be policy priorities. Overall, strengthening indigenous social and cultural networks is vital to enhancing the health and well-being of older adults.

## Data Availability

Raw data (such as interview transcripts) are not publicly available due to the privacy of participants. However, anonymized data are available upon reasonable request through the corresponding author.
